# Training needs for research in health inequities among health and demographic researchers from eight African and Asian countries

**DOI:** 10.1186/1471-2458-14-1254

**Published:** 2014-12-10

**Authors:** Joke Haafkens, Yulia Blomstedt, Malin Eriksson, Heiko Becher, Heribert Ramroth, John Kinsman

**Affiliations:** Centre for Social Research and Global Health (SSGH) and Amsterdam Institute for Advanced Labour Studies (AIAS), University of Amsterdam, Amsterdam, the Netherlands; Umeå Centre for Global Health Research, Epidemiology and Global Health, Department of Public Health and Clinical Medicine, Umeå University, Umeå, Sweden; Institute of Public Health, University of Heidelberg, Heidelberg, Germany

**Keywords:** Social determinants of health, Capacity building, Research personnel, Africa, Asia

## Abstract

**Background:**

To support equity focussed public health policy in low and middle income countries, more evidence and analysis of the social determinants of health inequalities is needed. This requires specific know how among researchers. The INDEPTH Training and Research Centres of Excellence (INTREC) collaboration will develop and provide training on the social determinants of health approach for health researchers from the International Network for the Demographic Evaluation of Populations and Their Health in Low- and Middle-Income Countries (INDEPTH) in Africa and Asia. To identify learning needs among the potential target group, this qualitative study explored what INDEPTH researchers from Ghana, Tanzania, South Africa, Kenya, Indonesia, India, Vietnam, and Bangladesh feel that they want to learn to be able to conduct research on the causes of health inequalities in their country.

**Methods:**

Using an inductive method, online concept-mapping, participants were asked to generate statements in response to the question what background knowledge they would need to conduct research on the causes of health inequalities in their country, to sort those statements into thematic groups, and to rate them in terms of how important it would be for the INTREC program to offer instruction on each of the statements. Statistical techniques were used to structure statements into a thematic cluster map and average importance ratings of statements/clusters were calculated.

**Results:**

Of the 150 invited researchers, 82 participated in the study: 54 from Africa; 28 from Asia. Participants generated 59 statements and sorted them into 6 broader thematic clusters: “assessing health inequalities”; “research design and methods”; “research and policy”; “demography and health inequalities”; “social determinants of health” and “interventions”. African participants assigned the highest importance to further training on methods for assessing health inequalities. Asian participants assigned the highest importance to training on research and policy.

**Conclusion:**

The identified thematic clusters and statements provide a detailed understanding of what INDEPTH researchers want to learn in order to be able to conduct research on the social determinants of health inequalities. This offers a framework for developing capacity building programs in this emerging field of public health research.

**Electronic supplementary material:**

The online version of this article (doi:10.1186/1471-2458-14-1254) contains supplementary material, which is available to authorized users.

## Background

Reducing health inequities, defined as the “avoidable inequalities in health between groups of people within countries and between countries” [1] has been an implicit or explicit objective of health policy in many countries and international organizations for decades [2, 3]. And yet, health inequities between and within countries are stubbornly persistent [4].

### Health inequities

Three measures are commonly used to describe health inequities: health disadvantages, i.e., inequalities between segments of populations or between societies; health gaps, i.e., inequalities between the worse–off and everyone else; and health gradients, i.e., inequalities across the whole spectrum of the population [5]. Recently, the work of the members of Commission of Social Determinants of Health (CSDH) of the World Health Organization (WHO) has shed a fresh light on the causes of health inequities and how best to address their reduction. In 2008, the Commission’s final report [4] provided ample evidence that the most powerful drivers of health and health inequities are the social conditions in which people are born, live, work and age and the health systems that are put in place, referred to collectively as the social determinants of health (SDH), and that these SDH are, in turn, influenced by “upstream” structural drivers: the nature and degree of social stratification in society as well as society’s norms and values, global and national economic and social policies, and national and local governance processes. The CSDH developed a number of conceptual frameworks that describe pathways by which these determinants can lead to health inequities [4–10]. Based on the accumulated evidence, the Commission made three major recommendations for actions that will be needed to reduce health inequities: 1) improve living conditions; 2) tackle the inequitable distribution of money, power and recourses that people need to lead a healthy life. The third overarching recommendation was the need to expand the knowledge base on the social determinants of health, to evaluate the action taken, and critically, to develop a workforce that is trained in identifying SDH. Many governments and international organizations have endorsed these recommendations [11].

The United Nations launched the Millennium Development Goals (MDG) in 2000 with a focus on improving the situation of the world poorest countries and reducing health inequities [12]. Recent statistics on the health-related MDGs, however, confirm the earlier findings from the CSDH [13, 14]. That is, even though low and middle income countries (LMIC) have made remarkable progress on a number of health-related MDG indicators (e.g. the reduction of under- five-mortality and the incidence of TB and Malaria), it is apparent that significant inequities persist between the world’s most advantaged and least advantaged countries and between populations within countries. Indeed, many low income countries will not be able to meet the MDG health objectives by the target date in 2015. Much remains to be done in the post-2015 period, particularly in the lowest income countries of sub-Saharan Africa and South Asia, and in those affected by conflict or high rates of HIV [13]. Based on the recommendations from the “global consultation on health in the post-2015 agenda”, it is to be expected that there will be a greater focus on the reduction of health inequities and SDH in the post-2015 development framework [15, 16].

To be able to address these issues through national or regional policies and programs, policy makers and other stakeholders will need setting-specific, timely, and relevant evidence on the relationship between health inequalities, SDH and health outcomes. Yet, this type of evidence is not readily available in LMIC’s in Africa and Asia. Research on the socio-economic drivers of health inequalities is an emerging field in these countries [17–19], and training possibilities for SDH research are limited [20]. Thus, there is a need for the development of capacity-building activities to enable such research [3, 20].

### INTREC

The INTREC project (INDEPTH Training and Research Centres of Excellence) was established with this concern in mind. The details of INTREC are described elsewhere (http://www.intrec.info). Briefly, the project is conducted by a six-institution consortium, with four partners from the “North” (Umeå University in Sweden; Heidelberg University in Germany; the University of Amsterdam in the Netherlands; and Harvard University in the USA) and two from the “South” (Gadjah Mada University in Indonesia and INDEPTH, the International Network for the Demographic Evaluation of Populations and Their Health in Low- and Middle-Income Countries). With its Secretariat in Accra, Ghana, INDEPTH is an expanding global network, currently with 52 Health and Demographic Surveillance Systems (HDSSs) field sites in 20 countries in Africa, Asia, and Oceania [21], (http://www.indepth-network.org).

INTREC aims to develop and provide a SDH-related training program for INDEPTH researchers, thereby allowing them to generate new country-specific evidence on associations between SDH and health outcomes. The training program also seeks to provide researchers with skills for making the research findings available to relevant decision makers in their countries. The INTREC training program is set within INDEPTH’s larger capacity building program, and training activities will be coordinated by two regional Centres, one in Africa (Ghana) and one in Asia (Indonesia), to facilitate South-South and North–South collaboration in SDH training and research.

In the initial phase, INTREC activities are focusing on INDEPTH researchers from four African countries (Ghana, Tanzania, and South Africa and Kenya) and four Asian countries (Indonesia, India, Vietnam, and Bangladesh). If the initial program is successful, training activities will be expanded to researchers from INDEPTH surveillance sites in other countries.

### Training needs

The target group for the INTREC training consists of professional researchers, each with their own specific background, experience, practice and work setting (HDSS) and culture. In recent years, several conceptual and methodological frameworks for assessing health inequalities and SDH were published [4–9, 11, 22–24]. These frameworks provide clear ideas about what an informed SDH-researcher may need to know and, consequently, what they should be taught. However, the literature on continued public health and medical education [24, 25], and adult learning [26] has noted that, in order to be effective, continued professional education must be relevant to the specific learning needs of the professionals and particularly to their current work situation.

With the overall purpose to develop a needs-based training program, we conducted an explorative study to identify what health and demographic researchers from INTREC's eight target countries feel that they need to learn in order to be able to conduct research on the causes of health inequalities in their own country. We used a bottom-up research methodology, “concept mapping”, that allowed participants to provide their own ideas about topics they deemed relevant for training and to mention as many ideas as they wished.

## Methods

Concept mapping is a mixed-methods approach that combines group processes (brainstorming, sorting and rating) that are commonly used in qualitative studies with statistical procedures (cluster analysis and multidimensional scaling) to help a group describe its ideas on a topic of interest in a structured way and to represent those ideas visually through a map [27–31]. Concept mapping is an inductive research methodology that has been used effectively to explore stakeholder perspectives across a range of fields [32–35], including education development [36]. While concept mapping was initially developed for use in face-to-face group sessions, software for web-based applications has become available in recent years. In this project we conducted a web-based concept-mapping exercise using Concept Systems Global Max software to support data entry and analysis [37].

### Participants

As is common in qualitative research designs, we used purposive sampling [38]. Our purpose was to recruit researchers who worked as scientists at one of the INDEPTH HDSSs in Ghana, Tanzania, South Africa, Kenya, India, Vietnam, Bangladesh, or Indonesia or at Gadjah Mada University in Indonesia, and who are familiar with the collection and analysis of household surveillance data that include information about health issues. We asked the INDEPTH Secretariat in Accra and the INTREC partner from Gadjah Mada University Indonesia to provide us with names of researchers who met these criteria. They provided names and addresses of 150 potentially suitable candidates. The project team sent all of them an invitation to participate in the concept mapping exercise by e-mail. The invitation included a web-address with which those who were interested could sign up to the project. After signing up, the study’s home page could be entered. The home page provided background information on the project and offered instructions on the various assignments could be answered.

### Data collection

Concept mapping starts with the formulation of a focal question. The project team developed the following ‘focus prompt’: “*In order to conduct research on the causes of health inequalities in my country, I would need background knowledge on*…”

Data were collected in two rounds. The first round consisted of a brainstorming exercise. Participants were asked to produce as many statements as they wished in response to the focus prompt, to keep their statements brief, so that they would express only one thought, and to type them in a textbox displayed in the concept mapping web application. Participants had about three weeks to respond to this request. After the brainstorming round was finished, the list of statements generated by the participants was reviewed by three members of the project team (JH, JK, YB) to eliminate duplicates and to edit the remaining statements to minimize any confusion in meaning. The goal was to have a set of mutually exclusive statements that express only one idea without loss of the original content. This set of statements was then used for the second round of data collection. In this second round, which started about three weeks after the brainstorming exercise was finished, participants were asked to rate the set of statements, on a five point scale, in terms of how important they felt it would be for the INTREC training program to provide instruction on the subject matter that is mentioned in the statement (1 not important at all, 5 very important). They were also asked to sort these same statements logically into thematic groups or clusters, using at least 2—but no more than 10— clusters and to provide a name for each cluster that covers its thematic content. Respondents had about one-and-a-half months to complete this second round of concept mapping.

In addition, all participants were requested to fill out a short questionnaire with eight questions concerning their background characteristics (institutional background, function, years of working experience, educational background and level, whether they thought their employer is interested in SDH, age and gender).

Data were collected between May and September 2012. For each round of data collection, invited participants who had not responded received two reminders from the research team supported by the INDEPTH Secretariat.

### Data analysis

To analyse the data, we used the Concept Systems Global Max package [37], which is based on the steps for the analysis of concept-mapping results that have been described in detail by Trochim and colleagues [27–29].

**Figure 1:**
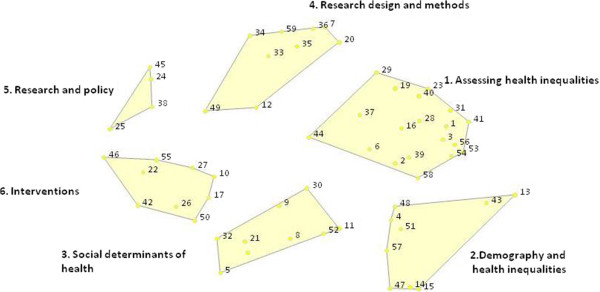
**Concept map of 6 clusters with labels and statements** ^**a**^ **, based on African and Asian researchers’ sorting results of 59 statements on what knowledge is needed to conduct research on the causes of health inequalities in their country.**

Analyses were performed for the whole group of respondents and separately for the subgroups of Asian and African researchers to explore any variations in opinion between the two groups. Participants’ results from the sorting activity were aggregated and analysed to identify how they categorized the statements that were generated during the brainstorming round. First, a similarity matrix was constructed that represents the relative similarity of participants’ sorted statements. Using multi-dimensional scaling with a two dimensional solution [31], the similarity matrix was then analyzed. The MDS analysis yielded a two-dimensional point map on which each statement is located as separate point on the map. Statements that are depicted closer together on this map have been grouped together more frequently by the participants. Subsequently hierarchical cluster analysis [30] was performed to identify relevant, interpretable and relatively homogeneous clusters of statements. This technique starts with each case (statement) in a separate cluster and then combines the clusters sequentially, reducing the number of clusters at each step, until only one cluster is left. This yielded a point-cluster map, on which the statements on the point map are now partitioned into groups of statement or clusters (see Figure 1 below). Each cluster is given a label or a name that summarizes the broader thematic content of the statements it contains. The cluster names that will be presented in this paper were selected by the research team, based on the names the participants had assigned to the groups of statements they sorted. The decision on how many clusters will be represented in this paper was also taken by the research team, considering both the contents and the potential meaningfulness of a thematic cluster for developing INTRECs educational program.

The aggregated importance rating scores the participants had assigned to the statements that were generated during the brainstorming round were used to calculate the average importance ratings participants had assigned to each statement. This resulted in a rated list of statements for all participants and for the subgroups of African and Asian researchers. The rating results were also used to calculate the average importance ratings of the sorted clusters. Rating scores indicate participants’ perceptions of how important it is that the INTREC training programs provides instruction on the subject matter referred to in the statement or in the cluster of statements.

Finally, to compare the average cluster ratings of African and Asian researchers, a pattern match or ‘ladder’ graph” was used. A pattern match is a bivariate comparison of the average cluster ratings that shows aggregate patterns of the ratings of -in our case- two groups of respondents. Instead of being arranged in typical *x*,*y* axis form, the two axes (one for each group) are set vertically side by side and joined by a separate line for each cluster that indicates average cluster ratings. A Pearson Product–moment correlation was calculated to describe the statistical correlation between the average cluster ratings of the two groups.

### Ethics

The ethical requirements of the Netherlands, the country of origin of the Principle Investigator (JH), applied to this study. This study used a social scientific method to explore opinions of researchers. It is not a medical study and participants were not subjected to medical procedures or required to follow rules of behaviour. Therefore, the study was exempt from review by the medical ethics committee of the Academic Medical Centre of the University of Amsterdam or any other authorized medical ethics committee in the Netherlands. This is in conformance with the Dutch Medical Research Involving Human Subjects Act (WMO). (See, http://www.ccmo.nl/attachments/files/ccmo-notitie-definitie-med-wet-onderzoek-25-11-05.pdf) However, by way of good research practice, the research team has followed the recommendations with regard to respect for human subjects involved in medical research from the research code of the Academic Medical Centre of the University of Amsterdam [38]. Concretely, invited participants were informed about the purpose, the procedures and implications of participating in the concept mapping study through an invitation letter. From those who decided to participate, written informed consent was acquired prior to participation. Participants were informed that confidentiality rules would be applied when storing, analyzing and reporting personal data and they were given the opportunity to withdraw their contribution at any time.

## Results

Of the 150 researchers who were invited for the study, 82 (55%) participated in at least one concept-mapping activity (brainstorming, rating, sorting). Their background characteristics are presented in Table 1. Fifty-four came from Africa and 28 from Asia. The group of African participants included researchers from 13 INDEPTH HDSSs in Ghana, Tanzania, South Africa and Kenya. The group of Asian participants included 18 researchers from 6 INDEPTH HDSSs in India, Vietnam, Bangladesh and Indonesia, as well as 10 researchers from Gadja Mada University, most of who had worked with surveillance data from an INDEPTH HDSS in Indonesia. With 81% of the respondents having an MA/MSc degree or higher, the educational level of the participants was typically high. The participants had a wide range of different educational backgrounds, e.g., medicine, social science, demography, computer science. This reflects the educational level and multidisciplinary character of the research teams working in INDEPTH HDSSs. Sixty-nine participants contributed statements in response to the focus prompt during the brainstorming round, 30 completed the sorting assignment and 41 rated the statements with respect to their importance for the INTREC training program. Twenty-nine participants completed all three assignments, 22 completed two of the assignments and 33 completed only one assignment.

**Table 1:** **Characteristics participants (N = 82)**

Characteristic		N	%
*Organisation*	African INDEPTH HDSS^*^	54	66
	Asian INDEPTH HDSS/University^**^	28	44
*Function*	Manager/Administrator	11	13
	Researcher	56	69
	Research assistant	4	5
	Lab worker	0	0
	Other	5	6
	Did not respond	8	9
*Working years*	Less than 2 years	0	0
	2-5 years	21	26
	5 years or longer	53	65
	Did not respond	8	9
*Educational background*	Medicine	19	23
	Nursing	1	1
	Social sciences	11	13
	Economics	2	2
	Agricultural sciences	1	1
	Demography	12	15
	Computer sciences or mathematics	12	15
	Other	16	20
	did not respond	8	9
*Educational level*	MD	5	6
	DSc/PhD	22	27
	Masters level	39	48
	Bachelors degree	7	9
	College or vocational degree	1	1
	did not respond	8	9
*Is employer interested in SDH?*	Yes	61	74
	No	0	0
	Not sure	12	15
	Not applicable	1	1
	did not respond	8	9
*Age*	18-30 years	13	16
	31-50 years	52	63
	51 years or older	9	11
	did not respond	8	9
*Gender*	Male	51	62
	Female	31	38

^*^Agincourt, Digkale, Africa Centre, Ifakara, Rufiji, Magu, Nairobi, Kilifi, Kisumu, Mbita, Navrongo, Dodowa, Kintampo.

^**^Vadu, Ballabargh, Matlab, Purworejo, Filabavi, Chililab, Gadjah Mada University, Indonesia.

### Statements

The brainstorming round yielded 108 statements. After the research team removed duplications these 108 statements were brought down to a list of 59 individual statements expressing the participants’ ideas on what background knowledge is needed to conduct research on the causes of health inequalities in their country (see Additional file 1). Table 2 lists the five statements with the highest ratings for African and Asian researchers and for the whole group, respectively. The rating scores indicate how important participants thought it would be for the INTREC training program to provide instruction on the subject matter referred to in the statement. The Table shows that there was a confluence of opinion between the Asian and African researchers. Two of the four highest rated statements were the same for both groups (translating research into policy, and analysis of longitudinal data). Statements referring to methods for measuring, monitoring and evaluating health inequalities were also deemed to be very important by both groups. In addition, instruction on health systems influencing health inequalities was perceived as important by African researchers, while Asian researchers emphasized the importance of instruction on community-based public health interventions.

**Table 2:** **Top five statements, rated in terms of how important** ^**a**^ **it would be for the INTREC training program to provide instruction to the topic referred to in the statement (African participants, Asian participants, all participants)**

Statement number (corresponds to list in Additional file 1)	Statement (topic)	Average importance score
**African researchers**		
23	Indicators to measure, analyse and evaluate (the dynamics of) health inequalities in different contexts	4.47
24	Translating research into policy: how to package lessons learned from research projects into policy messages	4.40
29	Methods for measuring/studying health inequalities	4.37
20	Analysis of longitudinal data	4.33
6	Health systems influencing health (in)equalities	4.23
**Asian researchers**		
1	Evidence on causes of health inequalities in my country	4.50
24	Translating research into policy: how to package lessons learned from research projects into policy messages	4.42
20	Analysis of longitudinal data	4.33
35	Monitoring and evaluation methods	4.33
22	Public health and public health interventions in the community	4.25
**All researchers**		
20	Analysis of longitudinal data	4.39
24	Translating research into policy: how to package lessons learned from research projects into policy messages	4.39
23	Indicators to measure, analyse and evaluate (the dynamics of) health inequalities in different contexts	4.37
29	Methods for measuring/studying health inequalities	4.24
22	Public health and public health interventions in the community	4.22

^a^1 = not important and 5 = very important.

### Identified themes for training

Separate analysis of the sorting results for African and Asian researchers revealed that both groups sorted the statements in rather similar sets of thematic clusters (data not shown). For that reason we only present the combined sorting results for all participants.

The concept map, representing participants’ ideas on what knowledge is needed to conduct research on health inequalities in their country, is shown in Figure 1. The Figure shows that the sorted statements could be grouped into a set of six distinct thematic clusters.

Table 3 describes the content of the six clusters: the overarching cluster theme, the individual statements (items) that are contained within each cluster, and the average importance ratings for each cluster and statement within the cluster. Overall, the Table indicates that participants assigned a rather high importance to each of the clusters, as ratings varied from 3.66 to 3.94. The two themes the researchers considered most important for an INTREC training program refer to methodological issues, namely: Cluster 1 - *assessing health inequalities* (3.94) and Cluster 4 - *research design and methods* (3.85). These clusters were followed by the somewhat lower ranked clusters; 5 - “*research and policy*” (3.84) and 2 - “*demography and health inequalities*” (3.77). Cluster 3 – *social determinants of health (3.67)*, and 6 – “*interventions*” (3.66) received the lowest average rankings. Taken together, the thematic areas and topics that are described in Table 3 offer an understanding and a framework of what health and demographic researchers may want learn to strengthen their ability for conducting research on determinants of health inequalities.

**Figure 2:**
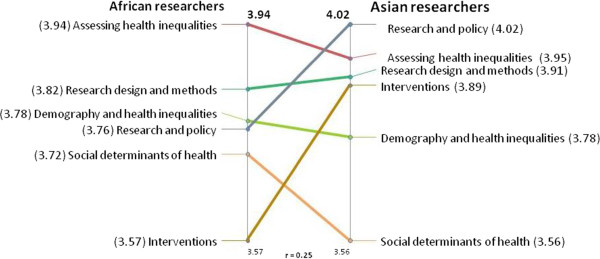
**Pattern match comparing the average cluster ratings from African and Asian participants in terms of how important it would be for the INTREC training program to provide instruction on the theme.**

The results of the pattern match that was performed to explore the level of agreement between African and Asian researchers on the relative importance of the six previously described thematic clusters for the INTREC training program are shown in Figure 2. The left vertical line in the graph shows the average cluster ratings from the African researchers and the right vertical line those from the Asian researchers. The lines drawn between the clusters allow us to compare how the average cluster importance ratings of the two groups connect. If the ratings of the two groups are in alignment the graph would resemble a ladder. Likewise, lines crossing at steep angels portray a lack of linkage between the priority ratings.

**Table 3:** **List of topics suggested by participants** ^**a**^ **, categorized into thematic clusters, with average priority ratings in terms of how important it would be for the INTREC training program to provide instruction the theme/topic** ^**b**^

***Cluster/Topic number***	***Themes and topics***	***Average Rating***
**1**	**Assessing health inequalities**	**3.94**
23^C^	Indicators to measure, analyse and evaluate (the dynamics of) health inequalities in different contexts	4.37
29	Methods for measuring/studying health inequalities	4.24
6	Health systems influencing health (in)equalities	4.17
2	Variation in access to health services for different groups	4.15
1	Evidence on causes of health inequalities in my country	4.1
3	The evidence on inequalities in health between the poor and wealthy	4.02
16	Concepts of disease and health inequality	3.98
44	Mapping the available health facilities and quality of health services they offer	3.95
37	Understanding methods that advance health equity in my country	3.95
19	Analysis of life-course as a cause of health inequalities	3.93
28	Theoretical background knowledge on the concepts of equity, inequalities, social determinants, and health inequities	3.93
56	Health inequalities: definitions, drivers, and means of addressing them	3.88
40	Systematic reviews of health inequalities	3.85
58	Rural–urban disparity in health service provision	3.83
54	Social and structural explanations of health inequalities based on characteristics of populations and effective interventions	3.8
31	Social network analysis as a means of mapping social and health inequalities	3.76
41	Discussion with experts about evidence/reviews on health inequalities	3.73
53	Consequences of impoverishment and income inequality arising out of high health care expenditures	3.61
39	How community members/local people in my country perceive and explain inequalities in health	3.61
**2**	**Demography and health inequalities**	**3.77**
13	Demography: demographic parameters and health inequalities	4.12
15	Demographic Changes	4
43	Population dynamics as factors affecting social inequalities in health	4
14	The demographic profile and economic profile of the population (and health)	3.95
57	Health profile of the country: the distribution of disease by age and sex	3.73
4	Health outcomes as a result of lifestyle differences within and between HDSS areas	3.71
48	Gender issues in relation to the structure and distribution of health services	3.68
51	Social injustice	3.51
47	Migration	3.25
**3**	**Social determinants of health**	**3.67**
9	Health (care) seeking behaviours	4.1
8	Environmental parameters that affect health	3.88
5	Health status of the elderly	3.8
52	Health transition	3.8
30	What are the wider social determinants of health (for example, education, employment, income, socio economic status, housing, gender)?	3.76
32	Children with special needs	3.32
18	Mid-life health concerns of men and women	3.29
11	Social exclusion and development	3.27
**4**	**Research design and methods**	**3.85**
20	Analysis of longitudinal data	4.39
35	Monitoring and evaluation methods	4.07
59	Qualitative research methods	4
7	Biostatistics	3.95
34	Writing research proposals and designs	3.95
36	(Advanced) statistical software and methods (e.g., for modelling)	3.93
12	(Social) epidemiology	3.88
33	Health economics and cost effectiveness studies	3.61
49	ICD 10	2.85
**5**	**Research and policy**	**3.84**
24	Translating research into policy: how to package lessons learned from research projects into policy messages	4.39
25	Health policy analysis (including decision-making process)	4.1
45	Research policy	3.54
38	Information about other people working in this field in my country	3.32
**6**	**Interventions**	**3.66**
22	Public health and public health interventions in the community	4.22
26	The relation between health policy and social determinants of health (access to care)	3.95
42	Health policies and politics as social determinants of health	3.76
10	Concept of health education/promotion (e.g. in the family, in migrating communities, by empowering women)	3.6
46	Health financing (incl. insurance)	3.54
27	The effect of subsidized or non-subsidized services on (population) health	3.51
50	Health awareness of decision makers in the family	3.49
17	Limited access to health information/education	3.46
55	Health infrastructure in my country	3.44

^a^Researchers from 13 INDEPTH HDSSs in Africa and from 6 INDEPTH HDSSs and a university in Asia.

^b^1 = not important and 5 = very important.

^c^Corresponds to cluster map composed of final list of 52 statements (see Figure 1).

The pattern shows that average cluster ratings of the two groups are close. There is relative accordance between both groups on the importance of three clusters: “*assessing health inequalities*”, “*research design and methods*” and “*demography and health inequalities*”. However, the researchers from Asia gave a higher priority to the clusters “*research and policy*” and “*interventions*”, than those from Africa. In contrast, African researchers assigned a higher priority to the cluster “*social determinants* This shows that the African researchers were somewhat more interested in receiving training on how to assess health inequalities (concepts and methodology), while Asian researchers were somewhat more interested in receiving training on how to address health inequalities (through policy or intervention research). The correlation coefficient (r = 0.25) is shown underneath the graph. This means that there is a-low-to-intermediate statistical association between average cluster ratings of the two groups.

## Discussion

In the years to come, more knowledge and analysis will be needed if we are to understand the relationship between SDH and health inequalities in LMIC’s, in order, thereby, to support the development of appropriate health equity policies [15]. Östlin et al. have characterized SDH research as “a paradigm shift” or a “new wave” in health research [10]. Continued professional training of the available cadre of researchers is one of the recommended strategies to facilitate the implementation of this new approach [3, 4]. Our study is unique, in that it elicited information on the perspectives of experienced health and demographic researchers from eight LMICs on what instruction may be needed to facilitate research on the causes of health inequalities in their own countries. All the more so, because these researchers are working with health and demographic survey data that are currently regarded as vital sources of health information for developing countries [10, 39].

By using an on-line process of concept mapping, we were able to solicit the views of 82 researchers from different regions and local communities in eight countries within a relatively short timeframe and at relatively low cost. We observed that the quality of our data was very similar to the quality of the data we collected through a face-to-face group-based concept mapping approach in an earlier project [34]. Given the nature of global health research (i.e., multinational partnerships conducting research across various countries), the limited resources for health research in LMIC (particularly related to SDH [40]), and the need to reduce our environmental footprint, we believe that this approach could also be interesting for other global health projects that aim to solicit opinions of participants across countries.

To our knowledge, no previously published studies have asked researchers in LMICs for their own thoughts on training needs for health equity and SDH, but we have found that the SDH approach advocated by the WHO’s Commission on Social Determinants of Health seems to resonate among the researchers who participated in this study. For instance, in line with the final report of the CSDH [4], several of the statements and thematic clusters that were generated by the participants highlight the importance of building up skills for collecting and analyzing evidence on the social determinants of health inequalities, as well as ensuring sufficient knowledge for translating the available evidence into policy. The work of CSDH and associated scientists also emphasizes the importance of developing theoretical and conceptual frameworks as a foundation for investigating or tackling SDH, (e.g. [6] and [41]). Yet, our data reveal that, in relative terms, the participants in this study considered instruction on theories and concepts less important than instruction on practical methodological tools. Surprisingly, the cluster titled “social determinants of health” was assigned a relatively low rating in terms of its importance for a training program. This emphasis on methodological issues is understandable as much of the daily work of researchers who work in HDSSs is related to the technical content of the research activity. Consequently, some of methodological toolkits for studying health inequity and health policy, that have been published in the context of the CSDH, may be particularly useful as instruction materials for the target groups of the INTREC training program [7, 8].

The study also suggests that adaptation of the training program to local needs may be relevant, as the pattern-match results revealed that, in contrast to African researchers, Asian researchers were more interested in receiving training on intervention studies and the translation of evidence for policy.

### Implications

Our study has several important implications:

1. The themes and items that have been generated through this concept mapping process have served as an important foundation for developing the modules and the content of the INTREC training program. As has been suggested by the study participants, the first three modules of the program give ample attention to methodologies and tools for assessing SDH, while the last two modules deal with the translation of research evidence on SDH for policy [42].
2. The program has been taught by teachers from the USA, Europe and Indonesia to researchers from INDEPTH HDSSs in 8 Asian and African countries in 2013 and 2014, and it will be evaluated in 2015. Although it is expected that training will motivate students to initiate and conduct SDH research, further studies will be needed to assess the effect of the training. Some of the themes and topics that are suggested by this study may serve as items for evaluation studies.
3. The INTREC training program is transferable, so that it can be taught by other (local) teachers. It will be inserted in regular capacity building programs of the Gadjah Mada University and the INDEPTH network. Moreover, part of the program consists of an on-line course that will be available for free on the internet.
4. Even though we studied SDH learning needs among a specific group of researchers in Africa and Asia, we have described, through this study, an approach to the participatory development of a training program that may serve as an example for other capacity building initiatives in LMICs and elsewhere in this emerging and challenging field [43] of public health research.

### Limitations

Some remarks about our methodological approach are needed here. First, even though every effort was made to invite and include researchers from INDEPTH HDSSs in Asia, the majority of INDEPTH researchers came from African-based HDSSs. It is possible that statements contributed during the brainstorming round are less relevant to Asian INDEPTH researchers. However, it should be noted that for the purpose of qualitative interview studies, a sample size of 15–20 persons is widely considered as sufficient [44]. After 15 cases, data saturation is usually reached and additional interviews will not offer conceptually new information [45]. We noticed that the phenomenon of data saturation also occurred during the brainstorming round, with new participants no longer adding new information to the list of statements that had already been generated by the other participants. For that reason, we can assume that the final list of 59 statements reflects the ideas of most of the participants quite well, including those of the Asian INDEPTH researchers.

Secondly, as has been the case in other online concept mapping studies [35], fewer participants in our study contributed to the rating and sorting activities than contributed to the brainstorm activity. This is probably due to the fact that these tasks are more time-consuming and complicated. Obviously, the perspectives of researchers who participated in all three activities of the concept mapping exercise weight more heavily on the final results than those of the ones who participated in only two or one activity.

Finally, it should be noted that the average cluster-rating scores were very close, varying from 3.66 to 3.94 for the whole group of respondents. In interpreting these results, it should be kept in mind that the concept-mapping method uses statistical techniques and computation mainly as a way to support the structuring of qualitative data, and not for making statistical inferences.

## Conclusions

The purpose of this study was to explore what health and demographic researchers from INTREC’s eight target countries feel they need to learn in order to be able to conduct research on health inequities in their country. The findings suggest a list of six thematic clusters that were deemed relevant for training: assessing health inequalities; research design and methods; demography and health inequalities; research and policy; social determinants of health; and interventions. Each cluster contains sub-topics that illustrate the content of the thematic clusters. Depending on the context in which researchers worked, there was some divergence of opinion about the relative importance of the different themes and topics. For instance, the African researchers saw instruction on how to assess health inequalities as a high priority for facilitating their future research on health inequities in their countries. The Asian researchers also assigned a high priority to instruction about how research evidence on SDH can be translated for use by policy makers, and on studying interventions that address the social determinants of health inequities. The identified themes and the topics offer a framework for developing a needs-based capacity building program for research for health equity and SDH.

## Electronic supplementary material

Additional file 1: **Statements generated in response to the brainstorm question.** (DOCX 16 KB)

## Abbreviations

CSDH: WHO’s Commission of Social Determinants of Health
HDSS: Health and Demographic Surveillance System
INDEPTH: International Network for the Demographic Evaluation of Populations and Their Health in Low- and Middle-Income Countries
INTREC: INDEPTH Training and Research Centres of Excellence project
LMIC’s: Low and Middle Income Countries
MDG: Millenium Development Goals
WHO: World Health Organization
MDS: Multi Dimensional Scaling
SDH: Social Determinants of Health.
