# Supplementary material for: Training needs for research in health inequities among health and demographic researchers from eight African and Asian countries
Source: BMC Public Health. 2014 Dec 10;14:1254. doi: 10.1186/1471-2458-14-1254 (PMC4295281; doi:10.1186/1471-2458-14-1254)
Supplement: Supplementary file 1 — Additional file 1: Statements generated in response to the brainstorm question. (DOCX 16 KB) [file 12889_2014_7373_MOESM1_ESM.docx]

**Additional file 1.**

**Statements generated by 69 health and demographic researchers from Asia and Africa in response to the focus prompt: In order to conduct research on the causes of health inequalities in my country, I would need background knowledge on...**

1. Evidence on causes of health inequalities in my country

2. Variation in access to health services for different groups

3. The evidence on inequalities in health between the poor and wealthy

4. Health outcomes as a result of lifestyle differences within and between HDSS areas

5. Health status of the elderly

6. Health systems influencing health (in)equalities

7. Biostatistics

8. Environmental parameters that affect health

9. Health (care) seeking behaviours

10. Concept of health education/promotion (e.g. in the family, in migrating communities, by empowering women)

11. Social exclusion and development

12. (Social) epidemiology

13. Demography: demographic parameters and health inequalities

14. The demographic profile and economic profile of the population (and health)

15. Demographic Changes

16. Concepts of disease and health inequality

17. Limited access to health information/education

18. Mid-life health concerns of men and women

19. Analysis of life-course as a cause of health inequalities

20. Analysis of longitudinal data

21. Nutrition and health behaviour

22. Public health and public health interventions in the community

23. Indicators to measure, analyse and evaluate (the dynamics of) health inequalities in different contexts

24. Translating research into policy: how to package lessons learned from research projects into policy messages

25. Health policy analysis (including decision-making process)

26. The relation between health policy and social determinants of health (access to care)

27. The effect of subsidized or non-subsidized services on (population) health

28. Theoretical background knowledge on the concepts of equity, inequalities, social determinants, and health inequities

29. Methods for measuring/studying health inequalities

30. What are the wider social determinants of health (for example, education, employment, income, socio economic status, housing, gender)?

31. Social network analysis as a means of mapping social and health inequalities

32. Children with special needs

33. Health economics and cost effectiveness studies

34. Writing research proposals and designs

35. Monitoring and evaluation methods

36. (Advanced) statistical software and methods (e.g., for modelling)

37. Understanding methods that advance health equity in my country

38. Information about other people working in this field in my country

39. How community members/local people in my country perceive and explain inequalities in health

40. Systematic reviews of health inequalities

41. Discussion with experts about evidence/reviews on health inequalities

42. Health policies and politics as social determinants of health

43. Population dynamics as factors affecting social inequalities in health

44. Mapping the available health facilities and quality of health services they offer

45. Research policy

46. Health financing (incl. insurance)

47. Migration

48. Gender issues in relation to the structure and distribution of health services

49. ICD 10

50. Health awareness of decision makers in the family

51. Social injustice

52. Health transition

53. Consequences of impoverishment and income inequality arising out of high health care expenditures

54. Social and structural explanations of health inequalities based on characteristics of populations and effective interventions

55. Health infrastructure in my country

56. Health inequalities: definitions, drivers, and means of addressing them

57. Health profile of the country: the distribution of disease by age and sex

58. Rural-urban disparity in health service provision

59. Qualitative research methods
